# L*a*b*Fruits: A Rapid and Robust Outdoor Fruit Detection System Combining Bio-Inspired Features with One-Stage Deep Learning Networks

**DOI:** 10.3390/s20010275

**Published:** 2020-01-03

**Authors:** Raymond Kirk, Grzegorz Cielniak, Michael Mangan

**Affiliations:** 1 Lincoln Centre for Autonomous Systems, School of Computer Science, University of Lincoln, Lincoln LN6 7TS, UK; 2 Sheffield Robotics, School of Computer Science, University of Sheffield, Sheffield S10 2TN, UK; m.mangan@sheffield.ac.uk

**Keywords:** fruit detection, deep learning, computer vision, agricultural robotics, multi-modal, strawberry perception, fruit localisation, outdoor detection, bio-inspired, one-stage networks

## Abstract

Automation of agricultural processes requires systems that can accurately detect and classify produce in real industrial environments that include variation in fruit appearance due to illumination, occlusion, seasons, weather conditions, etc. In this paper we combine a visual processing approach inspired by colour-opponent theory in humans with recent advancements in one-stage deep learning networks to accurately, rapidly and robustly detect ripe soft fruits (strawberries) in real industrial settings and using standard (*RGB*) camera input. The resultant system was tested on an existent data-set captured in controlled conditions as well our new real-world data-set captured on a real strawberry farm over two months. We utilise $F_{1}$ score, the harmonic mean of precision and recall, to show our system matches the state-of-the-art detection accuracy ($F_{1}$: 0.793 vs. 0.799) in controlled conditions; has greater generalisation and robustness to variation of spatial parameters (camera viewpoint) in the real-world data-set ($F_{1}$: 0.744); and at a fraction of the computational cost allowing classification at almost 30fps. We propose that the L*a*b*Fruits system addresses some of the most pressing limitations of current fruit detection systems and is well-suited to application in areas such as yield forecasting and harvesting. Beyond the target application in agriculture this work also provides a proof-of-principle whereby increased performance is achieved through analysis of the domain data, capturing features at the input level rather than simply increasing model complexity.

## 1. Introduction

The horticultural industry is facing many challenges due to its reliance on manual labour [1]. For example, soft fruit production involves many complex manual operations such as planting, plant care, yield prediction and fruit picking which remains difficult to automate. The first step in enabling automation in this application domain is a reliable and fast fruit recognition system which can provide the information about the presence, location and quality of the fruit [2].

**Figure 1:**
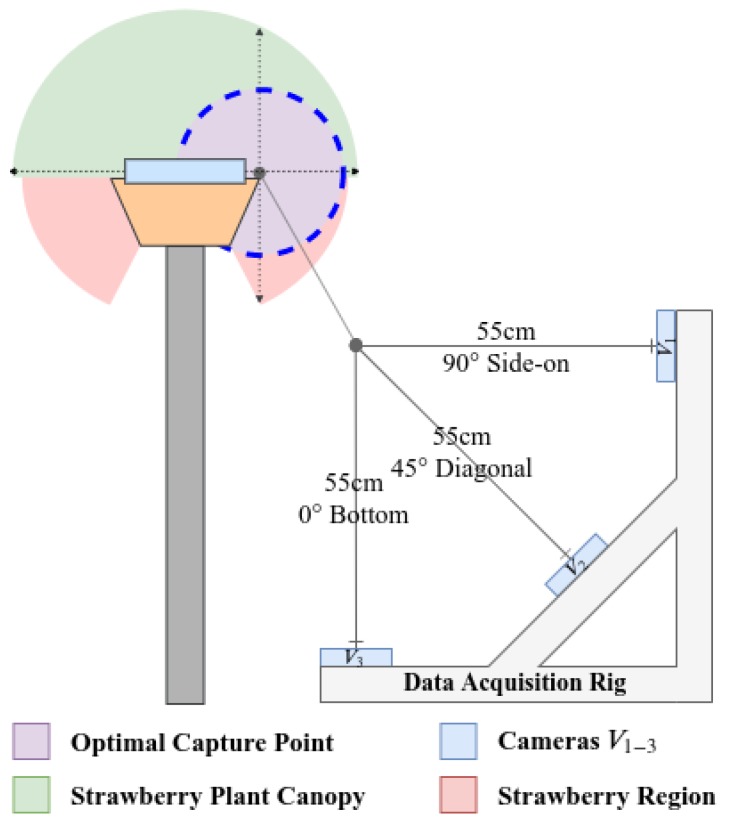
Camera configuration for viewpoints $V_{1}$, $V_{2}$ and $V_{3}$, where $V_{1-3}$ are camera identifiers.

**Figure 2:**
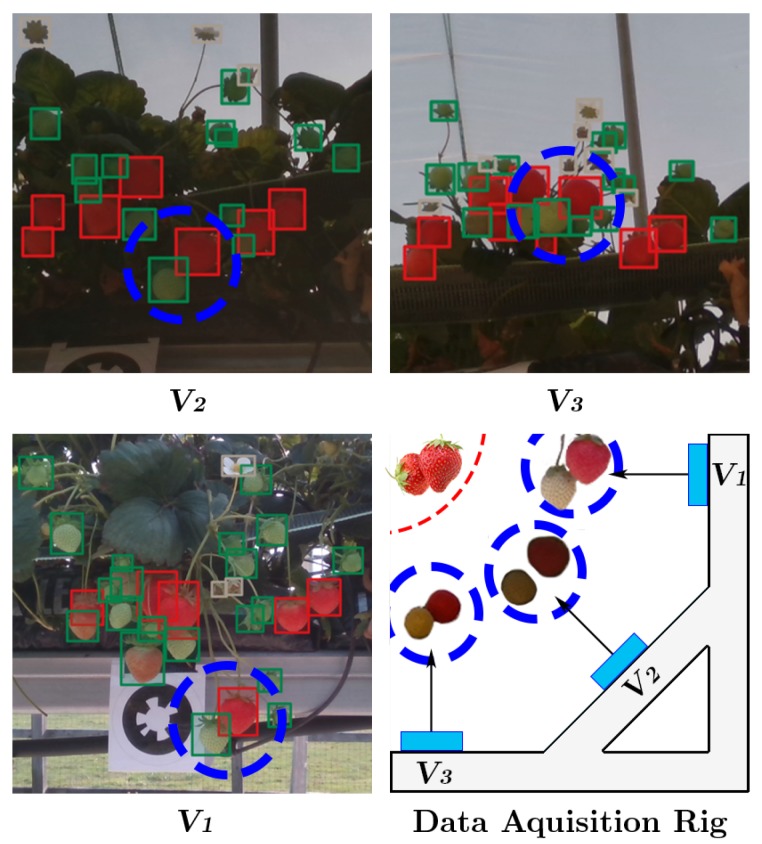
View point illumination variance for $V_{1}$, $V_{2}$ and $V_{3}$. Blue circles show the effect viewpoint has on class appearance.

Colour is one of the most relevant cues in detecting ripe soft fruit such as strawberries and shown to be directly related to their intrinsic attributes such as sugar level [3]. Yet, the visual appearance of fruit changes due to (a) different shape and texture between levels of maturity (b) variation of natural conditions such as weather, illumination, seasonal condition and growing cycles or (c) changes of camera viewpoint (see Figure 1 and Figure 2). Many approaches have shown promising results for classification, segmentation and localisation of crops [2,4,5,6,7]. However, as noted in [8], the problem of creating a fast and reliable fruit detection system still persists due to challenges described above.

The current state of the art in machine learning for object detection is represented by deep learning methods which have been also applied to agriculture with excellent results such as the DeepFruit network [8]. The network achieves very good performance but its robustness to natural variations is unknown since data acquisition in the presented work relies on heavily controlled lighting conditions (i.e. visible and near infrared LEDs in combination with a canopy) and the use of multi-modal sensing (*RGB* and Near Infra-red (*NIR*)).

The performance of any machine learning method, including deep neural networks, depends heavily on the quality and quantity of training data sets. Large data-sets do not currently exist for real-world agricultural setting and small data-sets will struggle to encompass all variations of parameters such as illumination. Therefore, the development in this field must look at alternate methods to boost performance and generalisation even when using small amounts of data.

In this work, we introduce a novel solution that moves beyond the state of the art by displaying greater invariance to environmental changes in the agricultural domain. Our high throughput fruit detection system utilises a combination of recent advancements in deep learning that have been shown to remove variance within data sets [9,10,11]. This approach incorporates an efficient feature extractor ResNet which fuses *RGB* and colour opponent data combined with a multi-scale feature pyramid network to deal with scale invariance and RetinaNet for classification with the modified focal loss function reducing class imbalance. We present an evaluation of this system on data sets collected from a real strawberry farm in natural conditions and compare its performance to the state-of-the-art network in Sa et al. [8]. The specific contributions of this paper are as follows:

1. Combining colour opponent features represented in *CIELab* space and *RGB* to provide greater multiple viewpoint invariance on networks trained on a singular view-point. This approach, referred to as early fusion, is then validated on viewpoints not present in the training data that show great variation in both spatial properties such as shape and illumination changes affecting colour.
2. Development of an accurate, high resolution and high throughput fruit detection system based on efficient network topology that can be trained on a low number of images in only one hour using state of the art approaches such as Feature Pyramid Networks [9], Residual Neural Networks [10] and RetinaNet [11].
3. Ablation study of the proposed system in Section 4. Showing the effect individual components of the system have on overall accuracy such as reduction of data set size and different permutations of model input.
4. Publication of an open access longitudinal strawberry data set captured in real agricultural environments from multiple views over a period of two months, each providing weather data, camera parameters, RGB, stereo infrared images and registered point clouds (available on https://github.com/RaymondKirk/labfruits_dataset).

This paper is organised as follows: a discussion of the related work in crop detection in agriculture and the application of deep learning methods is presented in Section 2 followed by a description of the proposed system (see Section 3). Section 4 presents the experiments used to validate our hypothesis of removing the effect luminance has on object detection through approximated human vision mechanisms. The paper concludes with a short summary and discussion of future work in Section 5.

## 2. State-of-the-Art

The automation of agricultural processes such as harvesting and yield prediction is the ultimate goal of recent research in vision systems for agricultural applications. Vision systems aim to segment, classify and localise fruit instances in the environment and provide meaningful semantic information such as area, position, size and maturity. Gongal et al. [12] review image processing techniques applied in to the agricultural domain, recent deep learning methods have outperformed classical image processing approaches and therefore represent the state-of-the-art (see recent review by Kamilaris et al. [13]). A recent approach by Sa et al. [8] applied standard deep-learning methods to fruit detection. Specifically, they use a two stage detector, Faster R-CNN [14], with a fusion of *RGB* and Infrared features to detect sweet peppers. For the late fusion approach, they train two networks on each of the image modalities and merge the results achieving an $F_{1}$ score (harmonic average of precision and recall [15]) of 0.838. However the fusion of these features doubles the complexity and resource requirement of their deep network, which is undesirable for time critical predictions in an agricultural environment. To address this issue they train a single network and combine the different image modalities at the input level instead. Using this early fusion approach halves the model inference time and achieves a $F_{1}$ score of 0.799 which is very comparable to their late fusion model performance. The data-set used however, does not consider illuminance variation, occlusion and colour similarity between crop and background classes. Which we attribute in this work as three major constraints limiting current approaches.

Roy et al. [16] tracked pomegranates over multiple frames, the authors note two distinct approaches to automatic robot harvesting, spectral-based and shape-based. Stating that spectral-based approaches are fast but weak to occlusions and inconsistent illumination whereas shape based are computationally expensive but more robust to these limitations. They obtain 96.6% accuracy with a 25.0% and 11.3% false positive and false negative error rate respectively by using K-Means clustering and morphological operations. The approach presented in [7] utilises super-pixel over-segmentation, dense SIFT descriptors and a bag-of-words histogram to classify fruits in images, achieving an accuracy of 97.6% for pineapples. A bag-of-words model was also used in [6] to find peppers in images in their two step automated fruit counting approach. Simple colour transformations and a naive Bayes classifier are used to detect initial regions of interest which are then in-turn used to train the bag of words model which uses texture and Maximally Stable Colour Region feature sets [17]. The estimates from multiple images are aggregated, limiting the impact of occluded fruits, to calculate the final fruit count. They note that a more comprehensive solution could have been achieved with 3D data. In [18] they combine both machine and deep learning based approaches for automatic observation of rice heading stage through a support vector machine and a convolutional neural network. Similarly to this paper they simplify the classification process by prior analysis and design of principled approaches.

Recent work within deep learning has shown great advancements in object detection and classification tasks [10,11,14,19,20]. The current approaches can be defined in two categories; one and two stage detectors. Two stage detectors are named so because they consist of (1) generating regions of interest using region proposal methods such as, BING [21], Selective Search [22], Region Proposal Network [14] and ContourBoxes [23], and (2) classifying and regressing the specified regions. One stage detectors on the other hand learn the bounding box location and classification labels simultaneously. Until RetinaNet [11] two stage detectors outperformed single stage detector accuracies; single stage detectors have higher throughput and consequently are much more desirable for real time applications. This achievement was contributed to the class imbalance problem one stage detectors generally face, where the background is too highly classified.

Two stage detectors such as Mask R-CNN [19] generate far fewer candidate locations than one stage detectors process and this was addressed by introducing a new loss function within RetinaNet to weight the cross entropy loss of these well classified classes (background) closer to zero. Recently hybrid approaches such as in [24] have been proposed using fast one stage approaches for easier detection problems and two stage detectors for robust detection on more difficult input data. They achieve this by estimating the difficulty of the input data before detection. In this paper we demonstrate the use of a one stage detector RetinaNet with a bio-inspired early fusion stage to combine the speed of one stage detectors, robustness to class imbalance shown by the architecture in [11] and detection at multiple scales through the introduced Feature Pyramid Network [9]. The motivation behind this approach is to remove variance observable in data sets such as scale and class imbalance at a higher, representational stage using colour. With this we bootstrap the model performance on multiple views the information already contained within the data set.

## 3. Materials and Methods

**Figure 3:**
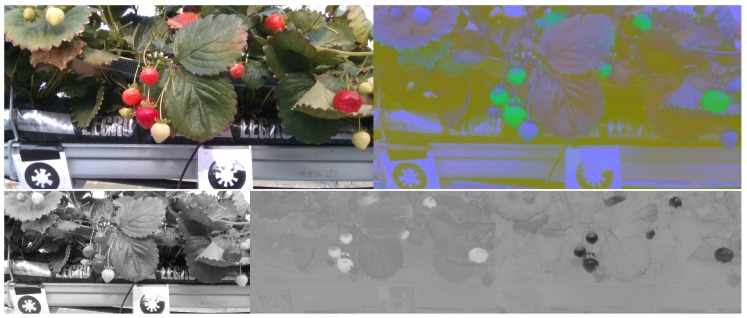
Network Input: Visualisation of *RGB* (**top left**) and *CIELab* (**top right**) used in model training. It’s evident in the opponent feature channels (**bottom row**) of *CIELab* that this colour space is appropriate for fruit detection due to the maximal and minimal response of fruit pixels.

**Figure 4:**
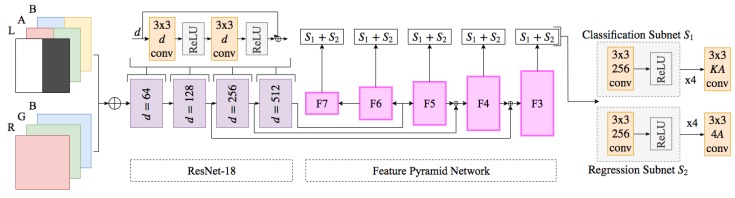
Model Architecture: RetinaNet implementation showing early fusion of *RGB* and *CIELab* features, where Fn are convolutional layers with resolution $2^{n}$ of the input and 256 channels. F6 and F7 are obtained by a 3×3 convolutional layer with stride 2 of F5 and 3×3 convolution with stride 2 and intermediate ReLU activation layer of F6 respectively.

The fruit detection system presented in this paper initially retrieves an image from either the data set (training) or the camera (testing). After the *RGB* data is captured and the transformation described in Section 3.1 is applied to convert into *CIELab* space. Figure 3 visualises the *RGB* data and *CIELab* data in the *RGB* colour space. The two images are then stacked depth-wise to form a D=6 dimensional tensor of size W×H×D where *D* is the dimensionality, *W* and *H* are the width and height respectively. At this point the fused image tensor is input into the network where a convolutional layer with stride 2 increases the dimensionality of the input *D* to 64 (chosen number of filters) via 2D convolutions of kernels of size 7×7. The ResNet-18 feature extractor then generates four feature maps from four blocks of a 3×3 convolution and ReLU activation function repeated twice at increasing number of input channels *D*. The latter three feature maps are then used in the feature pyramid network to generate five multi-scale feature maps, this process is visualised in Figure 4. For each scale created a classification and regression sub-net are applied. Respectively the sub-nets output tensors of size K·A and 4A where *K* are the classes and *A* are the predefined region proposals (anchors). In summary the classification sub-net outputs class predictions for each anchor and the regression sub-net outputs 4A regressed bounding boxes at each spatial location.

### 3.1. Colour Opponent Process

The approach presented in this paper is based on colour opponent process theory, network input features are modelled as an approximation of logarithmic function responses of photo-receptive materials in the human eye. Colour opponent process theory explains how the human vision system perceives colour information [25]. It explains colour vision as the combination of energy differences between opponent energy pairs. Red versus green, blue versus yellow and finally white versus black [26]. The first two opponent pairs model the perceived colour and the later opponent pair determines perceived luminance of an observed object. Simply, the opponent process is a translation between rod/cone responses to the combination of colours we perceive.

The motivation behind this is that luminance is contained entirely within a single opponent pair such that the three channels represent perceptually uniform colour, helping reduce one of the biggest constraints visions systems face; the impact of variable illumination in the environment. In computer vision the *CIELab* colour space approximates perceptually uniform human vision which means any change in the *CIELab* space should induce a similar change in the colour we perceive. *CIELab* has three channels each representing one of the colour spaces mentioned above, *L* represents white versus black, *a* represents red versus green and *b* represents blue versus yellow.

The opponent colour model has been applied in research numerous times [26,27,28,29,30], usually to tackle variable luminance. *CIELab* can naturally segment regions containing perceptually opposing colour channels, in [26] they state that objects have been designed/exist to be easily perceived by the human visual cortex. Things are described as easily perceived when the colour features maximally activate single components in each opponent pair. In example ripe and unripe strawberries both activate different ends of their respective red/green opponent pair. *CIELab* is used in [26] to detect the presence of roadworks without ever explicitly modelling any of the objects; traffic signs are usually bright oranges (yellow), blues and reds, corresponding to maximal activation of one component in each colour pair. *CIELab* extracts the visual saliency of colour features in objects and is used in research to model *RGB* more uniformly. As in [28] where it is used to generate colour models invariant of lighting and illumination changes.

The Deep Fruits system [8] attempts to solve the luminance problem by fusing multiple spectra, the visual *RGB* and infrared images. We aim to solve a similar problem by modelling the luminance through antagonistic colour pairs instead. The benefit of our approach is that it only requires *RGB* data from a standard camera and a non linear transform described in Equations (1)–(3) to convert between the two colour spaces. First to convert to the *CIE XYZ* space, described in Equation (1) where *Y* is modelled as luminance, *Z* is quasi-equal to blue stimulation, and and *X* is a linear combination of cone response curves. In Equation (1) the values used for *D* are calculated with regard to the *D65* illuminant [31].

(1) $$\begin{array}{c} \left[\begin{array}{c} X\\ Y\\ Z \end{array}\right]=\left[\begin{array}{c} D \end{array}\right]*\left[\begin{array}{c} R\\ G\\ B \end{array}\right]\\ D=\left[\begin{array}{ccc} 0.412453 & 0.357580 & 0.180423\\ 0.212671 & 0.715160 & 0.072169\\ 0.019334 & 0.119193 & 0.950227 \end{array}\right] \end{array}$$

Once *RGB* values have been transformed to *CIE XYZ* colour space a non linear transformation described in Equations (2) and (3) is applied to directly convert to *CIELab* space. In Equation (2) the values used for $X_{n}$, $Y_{n}$ and $Z_{n}$ are $X_{n}=95.047$, $Y_{n}=100.000$, $Z_{n}=108.883$ and are calculated under the *D65* illuminant [31] with normalisation $Y_{n}=100$. Note that this conversion from *RGB* to *CIELab* is device dependant and must be converted to the an absolute colour space such as *CIE XYZ* or *sRGB*.

(2) $$\begin{array}{c} \begin{array}{cc} L & =116f\left(\frac{Y}{Y_{n}}\right)-16\\ a & =500\left(f\left(\frac{X}{X_{n}}\right)-f\left(\frac{Y}{Y_{n}}\right)\right)\\ b & =200\left(f\left(\frac{Y}{Y_{n}}\right)-f\left(\frac{Z}{Z_{n}}\right)\right) \end{array} \end{array}$$

where f(x) adds the non-linearity and δ is equal to $\frac{6}{29}$.

(3) $$\begin{array}{c} \begin{array}{cc} f(x) & =\left\{\begin{array}{cc} \sqrt[{3}]{x}, & \mathrm{if}\;t>\delta^{3}\\ \frac{x}{3\delta^{2}}+\frac{4}{29}, & \mathrm{otherwise} \end{array}\right. \end{array} \end{array}$$

### 3.2. Early Feature Fusion

In our approach, we aggregate the selected colour spaces at the model input level and define our first layer of the model architecture accordingly. The first layer is defined as having *D* channels, where D=3 for either *RGB* or *CIELab* and D=6 for the early fusion model composed of both *RGB* and *CIELab* channels. A late fusion approach was also considered, which would combine predictions from two networks trained on *RGB* and *CIELab* inputs respectively but as found in [8] the performance gain is small and incurs linear increase of computational cost per network. In this case it was deemed that doubling the resources necessary for this small performance increase was an inefficient method of combining the information contained within each input transform domain.

### 3.3. Fruit Detection Using RetinaNet

Using the colour opponent process as model input attempts to maximise luminance invariance when training deep networks. Where variable luminance is a problem intrinsic to the data and contained classes, deep networks face other challenges. Some of the challenges are mitigated in this implementation through state-of-the-art approaches, such as ResNet-18 to help with the vanishing gradient constraint of deeper networks and Feature Pyramid Networks to mitigate issues with scale disparity between class samples in the training set. The combination of these approaches results in an architecture that can better learn features with high variation in luminance, spatial resolution (size of classes in input images) and intra-class balance (the ratio of class observations to other class observations, i.e., number of ripe strawberries objects in the training set to number of unripe strawberries).

#### 3.3.1. Feature Pyramid Networks

Detecting objects at multiple sizes and scales is a difficult problem in machine learning and has seen many different approaches in the computer vision domain. As mentioned above its unfeasible to construct a data set where the objective classes are well represented over all possible scales, illumination, shapes, colours and many other attributes. Such a data set would be need to be larger, meaning increased network training times and require infeasible levels of maintenance and annotation. One of the most popular recent advances in deep learning is Feature Pyramid Networks [9]. A image pyramid is comprised of multiple feature maps at different scales and are generally the output of sequential convolutional layers (i.e., an input image down sampled by a factor of 2, *n* number of times will create a feature pyramid where each layer is a different scale of the original down sampled image). Until recently this approach was mainly avoided due to the computational complexity and memory overhead they add to an architecture. To overcome the overhead, approaches have included using a single feature map from the feature pyramid, which looses the semantic information of the lower/higher layers or pyramidal feature hierarchies computed by sequential convolutional layers in a deep network [9]. However in these approaches there is a disparity between how semantically strong each layer is and therefor the effectiveness of this approach.

In [9] they exploit the inherent multi-scale, pyramidal hierarchy of deep convolutional networks to compute the Feature Pyramids at a much lower memory and computational cost while maintaining greater semantic information across each layer in the pyramid. The key contribution is the combination of lateral and top-down connections in the pyramid construction. Since lower level feature maps are not semantically strong the model will find it harder to learn from this information, generally deeper layers contain semantically strong information and are useful for classification/regression tasks. This approach uses top down connections so the model can learn as effectively or up to as well as the deepest layer containing the greatest semantic information. This process is described in much greater detail and clarification can be found for the terms in [9]. Using this Feature Pyramid Network in our approach helps maximise scale/size invariance while maintaining similar performance to using a single layer for feature extraction as mentioned above. In the original paper they increased the accuracy by 8.0% on the MS COCO data set [32] using this approach, for small objects generally missed, they increased the accuracy by 12.9%.

#### 3.3.2. RetinaNet

As discussed above the inclusion of the Feature Pyramid Network on top of the feature extractor we use, deemed ResNet [10], helps increase model performance over multiple scales. RetinaNet is a one-stage dense detector first presented in [11], the motivation behind this architectures development came from the fact one-stage detector performances were consistently trailing behind that of two-stage detectors such as Faster R-CNN [14]. The benefit of using one stage detectors is the speed, however until RetinaNet the speed generally cost model accuracy. The model accuracy loss was attributed to class imbalance during model training. To which they mitigated with the novel loss function they introduced deemed Focal Loss. This loss function reshapes standard cross entropy loss in a way that down weights well classified examples. With this new approach RetinaNet outperformed all two-stage detectors and matched the speed of one stage-detectors at the time of publication. We based our network architecture on RetinaNet to reduce the impact of class imbalance on network performance, this was key especially due to the data imbalance between ripe and unripe strawberries in the data set.

In our approach we use an 18 layer ResNet architecture with the discussed Feature Pyramid Network on top calculating feature maps at three scales from the ResNet-18 feature extractors basic blocks. For each scale we compute the probability that objects are present for each class *K* and at anchors *A*, and then regress anchor boxes *A* to nearby bounding boxes present in the ground truth. To achieve this we use two very similar sub-nets, a classification sub-net and a regression sub-net respectively. Composed of four 3×3 convolutional layers each with a ReLU activation layer attached. For the classification sub-net there is a final convolutional layer K·A filters and for the regression sub-net a 3×3 convolutional layer with 4A outputs. Both sub-nets have a final sigmoid activation layer attached to output binary predictions for K·A classifications and 4A regressed boxes per spatial location respectively. These sub-nets are described in greater detail in [11], where our implementation is based. Finally *A* number (9 in our approach) of boxes are generated at each location and focal loss for the regression and classification sub nets are calculated (using α=0.25, γ=2.00 in our approach). This constitutes the final loss as the sum of both classification and regression focal loss. The model architecture we used in our experiments is defined in Figure 4.

### 3.4. State of the Art Strawberry Detectors

In Table 1 we summarise the current state-of-the-art for deep learning based strawberry detectors. The columns in the upper table denote the number of images available in each data set, the availability of the data set, the camera viewpoint, whether the data set contains more than one imaging modality and finally if the data was captured in controlled (e.g., under controlled lighting) or natural agricultural conditions. The columns in the lower table denote the network architectures used, the average precision (used in the ImageNet challenge [33]), the $F_{1}$ score at an intersection over union of 0.5 and finally the inference speed (the time taken to generate predictions from a given input) at a specific image resolution.

We sought methods that were trained and tested with data-sets of comparable number of images and characteristics (viewpoint, multiple modalities, environmental conditions) allowing for fair and meaningful comparisons. Table 1 shows that closet approach with data that was freely available was Sa et al. [8], justifying its selection for comparison. While we do not compare with the remaining methods due to the inaccessibility of data-sets, Table 1 defines the performance scores and inference speed obtainable of different architectures on specific data sets. It is evident from the results that increased data set sizes and simpler viewpoints correlate to greatly improved accuracies, and it is difficult to compare different methodologies due to the variable complexity levels in the respective data-sets and their availability. to address this issue we provide a base line data-set (https://lcas.lincoln.ac.uk/owncloud/index.php/s/teQvy1iSLoUllU0) gathered in a real agricultural settings including multi viewpoints and modalities for future benchmarking studies.

## 4. Results and Discussion

In the following section we present our findings on reducing illumination and viewpoint variance on a challenging, real-world data set. We present (a) benchmark results for models trained on *RGB* data Section 3.1, (b) model results using the *CIELab* colour space, (c) model performance with early fusion of both colour spaces, (d) a evaluation of viewpoint (spatial) invariance between the three trained models describing a level of generalisation between unobserved views that alter the spatial appearance (shape, texture and colour of the class), and finally (e) a comparison to the Deep Fruits system [8] which similarly attempted to maximise illumination invariance but through multi-spectra fusion. Although we take it further and test our proposed solution on unseen views, Deep Fruits was found to be the closest baseline.

$F_{1}$ scores and the mean average precision metric used in the ImageNet challenge [33] are used in this paper to evaluate the experiments. The $F_{1}$ score is the harmonic average of precision *P* and recall *R* where precision is the number of true positives $T_{P}$ divided by the sum of true and false positives $F_{P}$, and recall is the number of true positives $T_{P}$ divided by the sum of true positives $T_{P}$ and false negatives $F_{N}$. The equation to compute the $F_{1}$ score using precision and recall is presented in Equation (4). An object is considered correctly detected in our results when the predicted bounding boxes have a intersection over union (IoU) of at least 0.5 (50%) with the ground truth annotation. However we also provide results using a value of 0.4 (40%) to enable more accurate comparison to the DeepFruits experiments. The justification provided for using the smaller intersection over union threshold in [8] is that objects in the data set are smaller than in the ImageNet challenge therefore require less overlap. We use values of 0.5 for non maximum suppression.

(4) $$P=\frac{T_{P}}{T_{P}+F_{P}},R=\frac{T_{P}}{T_{P}+F_{N}},F_{1}=2\cdot \frac{P\cdot R}{P+R}$$

### 4.1. Data Acquisition

Sa et al. [8] note that variation in outdoor agricultural environments affects vision systems greatly and many of the introduced factors such as sunlight and weather are detriment to the performance of machine vision systems. Current computer vision systems are either developed in controlled indoor conditions that avoid real-world constraints or use external equipment to minimise illumination variance in their data sets (see adaptation of data set from [38] by [8]). In this paper we present a longitudinal data set recorded in a real working agricultural environments containing RGB, stereo infrared images and point clouds as well as providing camera parameters, localisation and meta data describing capture conditions such as humidity and temperature. This data set was created in order to capture the variance present in natural outdoor conditions.

We captured 6189 images over 2 months, August and September 2018, and manually annotated 150 of them. Table 2 shows the number of images across each view that we used in the model training and validations (testing) stages. Note the data set used is small, as currently no large fruit data sets exist to the authors knowledge. All the Strawberries were labelled into two classes, Ripe and Unripe. The production site where the data was captured was at the University of Lincoln research farm at Riseholme campus. Two poly tunnels with table top strawberry rows were constructed, one row was tagged with visual markers [39] to indicate the points along the row where data should be collected, and the subsequent data collection process occurred singularly on this tagged row three times a day three times a week to capture various light intensities, weather conditions and plant growth stages. The species of strawberry was *Amesti*, captured at the flowering and fruiting stages of the plant.

**Figure 5:**
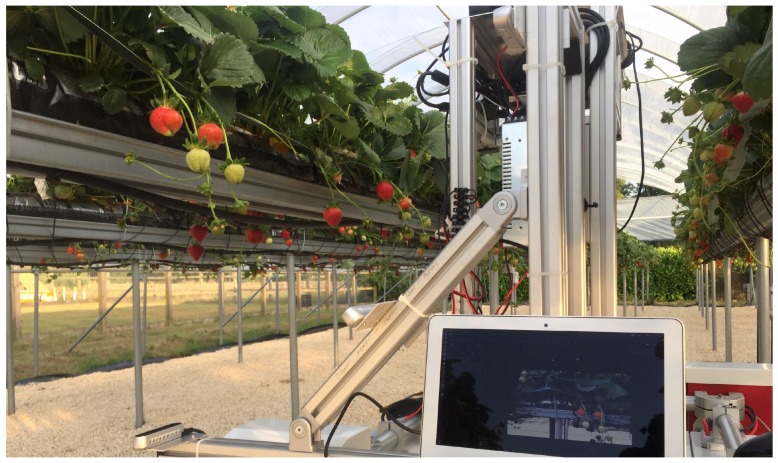
The image acquisition rig inside the strawberry polytunnels.

The images were captured at 1920×1080px resolution and the network was trained without resizing them. The data acquisition rig is visualised in Figure 2 and shown in Figure 5, three cameras were mounted 45 degrees apart to capture as much spatial information from the strawberry crops as possible. The top, middle and bottom cameras will each be referred to as $V_{1}$, $V_{2}$ and $V_{3}$ respectively from here on in. Capturing at these three distinct points ensured the information captured by each camera would have a good spread of dissimilar semantic information about each class. For example $V_{1}$ and $V_{3}$ would contain visually very different information for each class, whereas $V_{2}$ would share a greater instance information about each class with viewpoints $V_{1}$ and $V_{3}$; Shown in Figure 2. This enabled us to compare the impact viewpoint variance had on model performance. Each class could be trained on a training set that contained information from each viewpoint $V_{1-3}$, however we exclusively set the experiments up so only $V_{1}$ would be used in the model training stage. We did this to simulate the real-world effect of illumination, shape and texture changes introduced by unpredictable viewpoint variations caused by indeterministic environmental effects such as weather and human interaction.

During data collection the acquisition rig was mounted on a modular robotic platform Thorvald [40] and moved incrementally to each visual marker to ensure consistency between data collection cycles.

The data set presented should be considered a complex data set in the sense it contains classes with heavy occlusion and highly varied illumination. The images were captured over a period of 24 days with a intra-day variance of 11 hours. It contains two classes: Ripe Strawberry and Unripe Strawberry with uneven distribution as shown in Table 3. The difficulty of the data set is reflected in the quantitative assessment later in this paper. The data set will be made publicly available, in order to support key advances in this research area.

### 4.2. RGB and Early Fusion Comparison

In order to determine the effect of perceptually uniform colour spaces on viewpoint invariance we conducted three different experiments. The original motivation of this approach is that error due to variation in luminance of each class described in Section 4.1 would be minimised. To test this the *CIELab* colour space was used in order to capture the colour feature components present in the image more uniformly and thus fortify the features learnt in the network. We utilise an early fusion method, introduced in [8]. A late fusion method that combined two separate models was also proposed but we determined that doubling the number of parameters present in the network, computation time and GPU utilisation was an insufficient method to deal with luminance for reaching greater viewpoint performance.

As shown in Table 4 in terms of $F_{1}$ the early fusion approach outperforms both *RGB* and *CIELab* by 2.4% and 8.2% respectively on $V_{2-3}$. On the unseen viewpoints the $F_{1}$ score is lower as was expected since no images from either of these orientations was included in the training data set. The early fusion $F_{1}$ score for $V_{2-3}$ is 4.3% less than the result on the singular view $V_{1}$, the small difference in scores compared to the 6.4% and 8.8% drop for *RGB* and *CIELab* show that this approach can better generalise to unseen views of the each of the classes.

**Figure 6:**
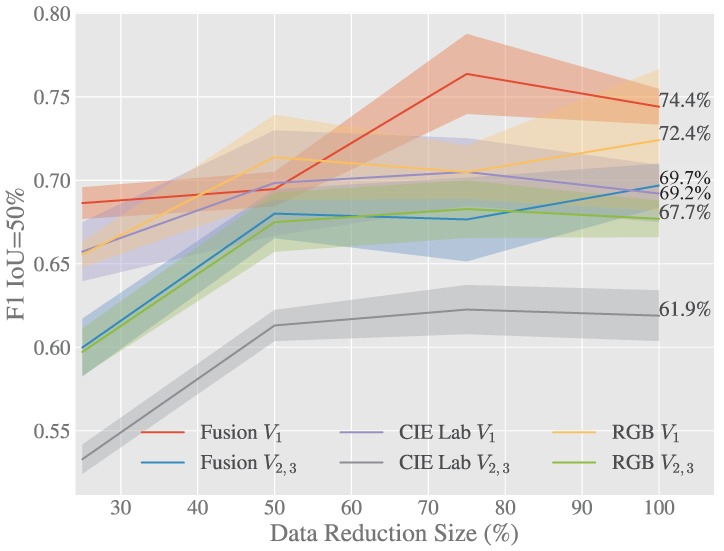
$F_{1}$ Score for 50% intersection over union on $V_{1}$ and $V_{2}+V_{3}$ validation data sets.

It can be seen in both Table 4 and Figure 6 that *RGB* and *CIELab* are both consistently outperformed by the early fusion method, while the fusion of these two features shows a great improvement over a singular approach alone. It’s also evident from this table that the lesser opponent class “Unripe Strawberry” has higher performance in *RGB* space however is still beaten by early fusion when on unseen views. The early fusion approach demonstrates greater invariance to luminance and achieves excellent results on previously unseen views described in Figure 2.

**Figure 7:**
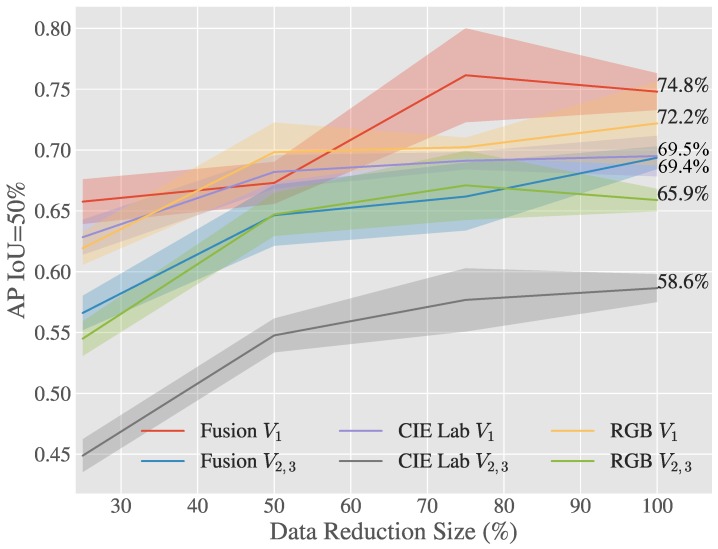
Average Precision for 50% intersection over union on $V_{1}$ and $V_{2}+V_{3}$ validation data sets.

**Figure 8:**
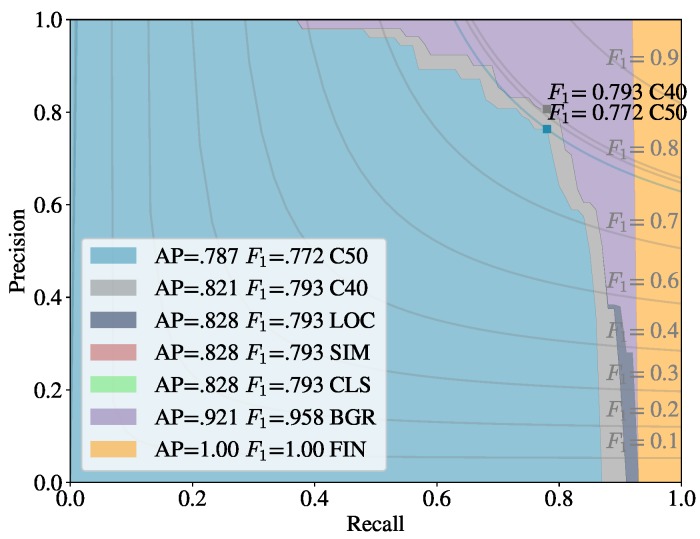
Precision-recall (PR) analysis: PR curves of the trained Sa et al. [8] early fusion network. C50, C40 and LOC correspond to PR curves for IoU values of 0.5, 0.4 and 0.1, LOC when all localisation errors are removed. SIM and CLS when errors from similar categories and all classification labels are removed. BGR is the PR curve when all other class/background false positives are removed and finally FIN shows PR containing no errors.

**Figure 9:**
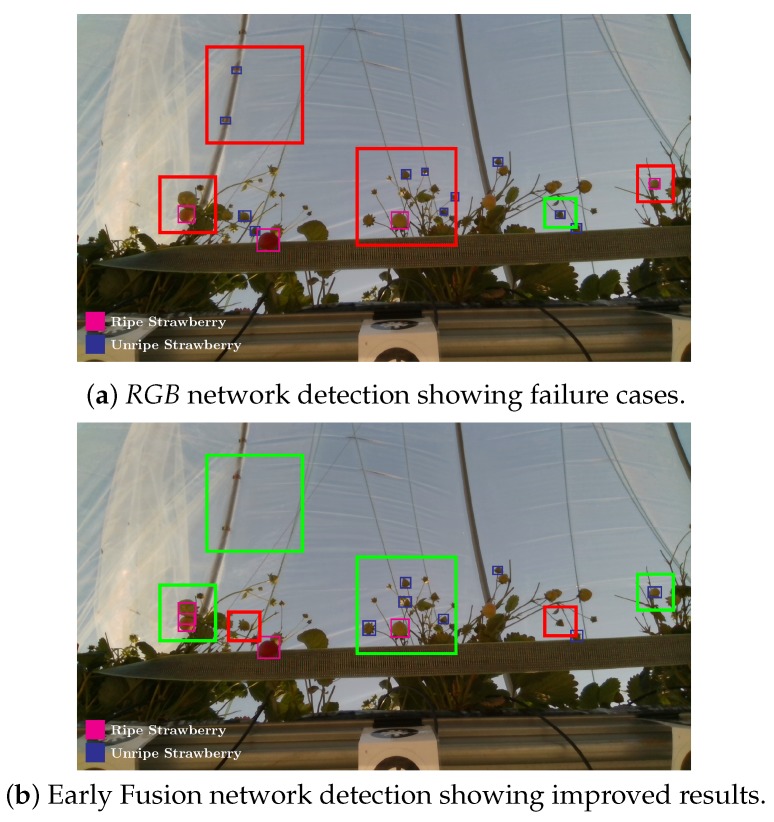
Performance on difficult input: Early Fusion and *RGB* models evaluated on $V_{3}$, the view with the highest spatial variation. The early fusion approach maintains detection accuracy over huge illumination and shape alterations (introduced from viewpoint). Improved results are shown in green and detrimental results shown in red.

Figure 6 and Figure 7 show how the Early Fusion approach responds to reduced data sizes. The original data size of 120 images described in Table 2 is a small data set and in the experiments shown in these figures it was reduced by a factor of 25%, 50% and 75%. Its observed from the gradient of the line that the methods evaluated may perform better when more data is available. It can be seen that for a heavily reduced data set the methods have much lower performance than they do with full data access. Data augmentation could be further used to boost the performance of early fusion by altering the luminance values within the fused data to uniformly alter colour features in the original *RGB* data. Figure 8 shows a false positive analysis of the early fusion network on the DeepFruits data set, you can clearly see in this graph most of the models inaccuracy comes from misclassification (BGR on Figure 8), data augmentation is one approach that could help minimise this issue. In Figure 9 we provide the output of our early fusion approach compared to *RGB* alone, highlighting the cases where it surpasses baseline performance. The results shown are from a network trained on $V_{1}$ data and evaluated on the most dissimilar view $V_{3}$ to stress the detectors performance across high variation of shape and luminance.

### 4.3. State-of-the-Art Comparison

The Sa et al. [8] paper noted the crucial component of autonomous fruit harvesters to be an accurate vision system, to which they attributed illuminance variation, occlusion and colour similarity between crop and background class to be the three major constraints limiting current approaches. They proposed a system based on the two stage detector Faster R-CNN that utilised early and late fusion of *RGB* and near Infrared imagery. Early fusion was a singular model with four input channels (*RGB* + *IR*) and late fusion trained two separate models (*RGB*, *IR*) and combined the detected objects in the final stage. Sa et al. [8] found late fusion to be the best approach achieving an $F_{1}$ score of 0.838 for Sweet Peppers, however they also noted this approach requires double the number of network parameters, computational cost, power, GPU utilisation, training time and inference time. Ultimately concluding the small decrease in accuracy of the early fusion approach from 0.838 to 0.799 as a worthy trade-off.

We compare our approach using a one stage detector RetinaNet to theirs in the following section. We directly compare the performance of three of our networks trained on RGB, *CIELab* and Early Fusion inputs to their Late and Early fusion approach. We compare the effectiveness of our perceptually uniform features *CIELab* to that of IR to remove luminance variance within the data set, described in Table 5. The evaluation metrics used are in correspondence with the original paper (IoU at 0.4) and only classification scores greater than 0.9 are considered. The images contained in the sweet pepper data set were not as high resolution as with our Strawberry data set described in Table 2 but instead were 1296×964px which we sampled to be divisible by 32 at 1280×736 pixels.

In this experiment we would expect lower performance values due to the fact we’re using a one stage detector over the two stage detector used in the original paper, as well as working with a data set less colour centric than ours. It is less colour centric due to the single class sweet pepper sharing very similar colour features with the background class. However we show the effectiveness of our approach at achieving what the addition of IR tried to achieve in DeepFruits, fortifying the prior viewpoint experiment results and luminance removal even when classes share much of their colour features that *CIELab* is based upon.

Moreover the early fusion approach attempted in the deep fruits paper failed at surpassing the $F_{1}$ score of standard *RGB* and their approach using late fusion (which did outperform the *RGB* baseline) was dependant on simultaneous collection of IR data as well as training two separate networks, ultimately only showing a 2.2% increase.

**Figure 10:**
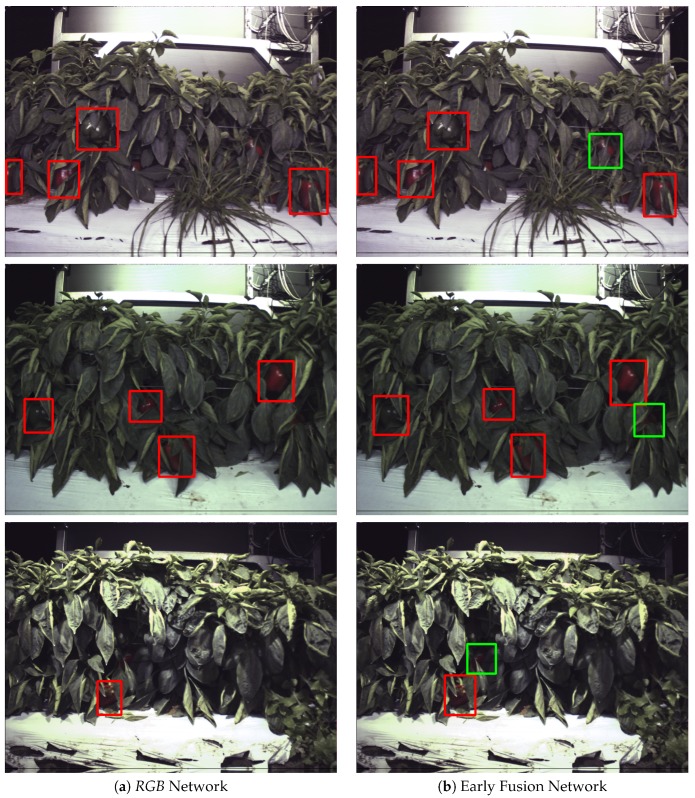
DeepFruits evaluation: Early Fusion (right) and *RGB* (left) models evaluated on the DeepFruits Capsicum data. It can be seen that the early fusion approach more frequently detects objects the *RGB* network misses (highlighted in green).

We observe similar results in Table 6, our early fusion approach closely follows the *RGB* $F_{1}$ scores and matches the performance obtained by Sa et al. [8] (0.799). Our average precision scores outperform the standard *RGB* results by 2.8% and 6.2% for AP50 and AP40 respectively, suggesting the network more accurately classifies than it detects. Although we show statistically similar results to DeepFruits, our approach is considerably faster (6.6×) at 0.06 s per image compared to 0.393 s and only needs *RGB* data instead of the *RGB* + Near Infrared data their approach requires. Interestingly the early fusion approach maintains similar precision increases over the experiments as in Table 4 and Figure 7. In Figure 10 we compare our early fusion approach to standard *RGB* on networks trained from the data provided by Sa et al. [8] originally captured in [38]. The results provided are representative of the results presented in Table 6.

Across experimental conditions we see consistent improvement in viewpoint invariance using early fusion of *RGB* and CIE Lab. Utilising the RetinaNet architecture as a base allowed us to remove class imbalance through the Focal Loss function and improve detection for objects at multiple scales through the implemented Feature Pyramid Network. We achieve near real time performance as seen in Table 7, where we present speeds similar to what is stated as near real-time in relevant literature [20]. Our early fusion approach adds to the architecture by providing results less sensitive to colour specificity of trained classes, and can be seen as a more generalised approach to solving this problem than introducing multiple spectra as in [8].

## 5. Conclusions

We present in this paper an example of improving network performance on unseen data through a structured approach and analysis of the network input. We chose a fusion of features instead of modifying network architecture and depth to increase generalisation to non-representative images. The results observed indicate that using bio-inspired features can avoid increased model complexity for increases in accuracy and generalisation capabilities. For colour centric data classes we conclude that this approach shows great promise in increasing the robustness of trained deep networks in real world conditions. The addition of *CIELab* helps increase viewpoint invariance by training on more specific colour features across a wider luminosity range within each class. With the introduction of multiple viewpoints or unknown viewpoints the environmental factors contributing to the appearance of objects in a scene change and *CIELab* provides a more normalised representation of each class when they’re colour centric (maximally activate a single component in colour opponent pairs). We achieve a 2.4% and 8.2% increase with our early fusion approach on unseen viewpoints $V_{2-3}$ over the standard *RGB* and *CIELab* modalities alone. In comparison the standard *RGB* and *CIELab* drop by 6.4% and 8.8% respectively for $F_{1}$ scores between viewpoints $V_{1}$ and $V_{2-3}$. Similarly when applied to the DeepFruits data set we gain an AP score increase of 2.8% (IoU = 0.5) and 6.2% (IoU = 0.4) over *RGB* alone. Our $F_{1}$ scores match those presented in the original paper, suggesting the added *CIELab* opponent features assist in classification of the detected objects more so than aiding the initial detection since our obtained AP scores are consistently higher than *RGB* in all cases (2.8% and 6.2%). Our approach also gains a performance increase of 6.6 times that of the DeepFruits early fusion method utilising IR and only considering a single class. This improvement is likely to increase the applicability of the method to robotic fruit monitoring and harvesting systems that have limited computational and power resources.

### Future Work

Leveraging *CIELab* colour opponent features with *RGB* helped mitigate some luminance variation in the validation sets. As can be seen in Figure 6 and Figure 7 the early fusion approach appears to improve with larger amounts of data. Investigation into the benefits provided by this approach as the data set size increases would provide insight to the limitations and optimal accuracy increase through our proposed methods. As well as calibrating the cameras to improve the colour accuracy over multiple sensors. Visualisation of features and filters learned in the network would also provide intuition as to what the network is learning which would be useful in seeing the difference between learnt *RGB* filters and colour opponent filters. To validate the removal of luminance further this analysis could compare network activation for synthetically created Strawberries at variable luminosity, where uniform activation over variable parameters would indicate the removal of the detrimental effects of the parameter on overall accuracy. Finally analysis into accuracy increase with fewer classes or binned classes would show whether error is introduced through learning multiple classes, due also to the fact this paper compares to Sa et al. [8] which noted multi-class detection as further work than the scope of the paper. 

## Figures and Tables

**Table 1:** Summary of SOTA (state-of-the-art) approaches to Strawberry Detection in Deep Learning.

**Method**	**# Images**	**Availability**	**Viewpoint**	**Multi Spectra**	**Controlled**	**Natural**
Yu et al. [34]	1900	✗	Side on (Close)	✗	✓	✗
Chen et al. [35]	12526	✗	Aerial	✗	✗	✓
Lamb and Chuah [36]	4550	✗	Ground	✗	✗	✓
Ge et al. [37]	-	✗	Side on	✗	✗	✓
Sa et al. [8]	122	✓	Side on	✓	✓	✗
L*a*b*Fruits (Ours)	150	✓	Multiple	✓	✗	✓
**Method**	**Network**		**AP (IoU 0.5)**	$F_{1}$ **(IoU 0.5)**	**Inference Speed (s)**	
Yu et al. [34]	Mask R-CNN - ResNet-50		-	-	0.13 @ 640×480px	
Chen et al. [35]	Faster R-CNN - ResNet50		0.77	-	0.11 @ 480×380px	
Lamb and Chuah [36]	Single Shot Detector (SSD)		0.84	-	0.61 @ 360×640px	
Ge et al. [37]	Mask R-CNN - ResNet-101		0.81	0.90	0.62 @ 640×480px	
Sa et al. [8]	Faster RCNN - VGG-16		-	0.79	0.39 @ 1296×964px	
L*a*b*Fruits (Ours)	RetinaNet, ResNet-18		0.75	0.75	0.07 @ 1920×1080px	

**Table 2:** Distribution of images across training and testing sets for $V_{1}$, $V_{2}$ and $V_{3}$.

Viewpoint	Training	Validation	Total
$V_{1}$	120 (80%)	10 (6.6%)	130
$V_{2}$	0 (0%)	10 (6.6%)	10
$V_{3}$	0 (0%)	10 (6.6%)	10
Total	120 (80%)	30 (20%)	150

**Table 3:** Distribution of labelled classes across training and testing sets for $V_{1}$, $V_{2}$ and $V_{3}$.

Bounding Boxes	Ripe	Unripe	Total
Training	673	2680	3353
Validation	217	649	886
Total	890	3329	4219

**Table 4:** $F_{1}$, Average Precision (AP) and Average Recall (AR) scores of *RGB*, *CIELab* and Early Fusion at 50% intersection over union (IoU) for each detected class. Bold indicates the best result in each row.

Class	View	Score	RGB	CIE Lab	Early Fusion
Both Classes	$V_{1}$	$F_{1}$	0.744	0.710	**0.747**
Both Classes	$V_{1}$	AP	0.722	0.695	**0.748**
Both Classes	$V_{1}$	AR	0.870	0.844	**0.909**
Both Classes	$V_{2-3}$	$F_{1}$	0.680	0.622	**0.704**
Both Classes	$V_{2-3}$	AP	0.659	0.586	**0.694**
Both Classes	$V_{2-3}$	AR	0.812	0.761	**0.851**
Ripe Strawberry	$V_{1}$	$F_{1}$	0.683	0.625	**0.697**
Ripe Strawberry	$V_{1}$	AP	0.616	0.571	**0.678**
Ripe Strawberry	$V_{1}$	AR	0.807	0.767	**0.892**
Ripe Strawberry	$V_{2-3}$	$F_{1}$	0.697	0.662	**0.729**
Ripe Strawberry	$V_{2-3}$	AP	0.659	0.621	**0.719**
Ripe Strawberry	$V_{2-3}$	AR	0.806	0.777	**0.877**
Unripe Strawberry	$V_{1}$	$F_{1}$	**0.805**	0.795	0.797
Unripe Strawberry	$V_{1}$	AP	**0.828**	0.819	0.818
Unripe Strawberry	$V_{1}$	AR	**0.933**	0.922	0.927
Unripe Strawberry	$V_{2-3}$	$F_{1}$	0.663	0.582	**0.679**
Unripe Strawberry	$V_{2-3}$	AP	0.658	0.552	**0.668**
Unripe Strawberry	$V_{2-3}$	AR	0.819	0.745	**0.825**

**Table 5:** Distribution of training and testing images used in DeepFruit models.

Class	Train	Validation	Total
Sweet Pepper (Capsicum)	100 (82%)	22 (18%)	122

**Table 6:** $F_{1}$ scores of *RGB*, *CIELab* and Early Fusion at AP40 and 50 on the DeepFruit data set (0.799 at AP40). Bold indicates the best result in each row.

IoU	Metric	RGB	CIE Lab	Early Fusion
40%	$F_{1}$	0.789	0.763	**0.793**
40%	AP	0.759	0.758	**0.821**
50%	$F_{1}$	**0.789**	0.738	0.772
50%	AP	0.759	0.705	**0.787**

**Table 7:** Performance of the Early Fusion Network on a Nvidia GTX 1080 Ti 11GB (single forward pass).

Resolution	Model Inference Time	Frames Per Second
1920 × 1080	0.073 s	13.71
1280 × 736	0.038 s	26.33
